# A translational study on looming-evoked defensive response and the underlying subcortical pathway in autism

**DOI:** 10.1038/s41598-017-15349-x

**Published:** 2017-11-07

**Authors:** Yu Hu, Zhuoming Chen, Lu Huang, Yue Xi, Bingxiao Li, Hong Wang, Jiajian Yan, Tatia M. C. Lee, Qian Tao, Kwok-Fai So, Chaoran Ren

**Affiliations:** 1 Guangdong-Hongkong-Macau Institute of CNS Regeneration, Ministry of Education CNS Regeneration Collaborative Joint Laboratory, Jinan University, Guangzhou, 510632 China; 2 Guangdong key Laboratory of Brain Function and Diseases, Jinan University, Guangzhou, 510632 China; 3 The Center of Speech Language Disorder, The First Affiliated Hospital of Jinan University, Guangzhou, 510630 China; 4 Laboratory of Neuropsychology, The University of Hong Kong, Hong Kong, China; 5 Laboratory of Cognitive Affective Neuroscience, The University of Hong Kong, Hong Kong, China; 6 State Key Laboratory of Brain and Cognitive Science, The University of Hong Kong, Hong Kong, China; 7 Psychology Department, School of Medicine, Jinan University, Guangzhou, 510632 China; 8 Department of Ophthalmology, The University of Hong Kong, Hong Kong, China; 9 Co-innovation Center of Neuroregeneration, Nantong University, Nantong, 226001 China

## Abstract

Rapidly approaching objects indicating threats can induce defensive response through activating a subcortical pathway comprising superior colliculus (SC), lateral posterior nucleus (LP), and basolateral amygdala (BLA). Abnormal defensive response has been reported in autism, and impaired synaptic connections could be the underlying mechanism. Whether the SC-LP-BLA pathway processes looming stimuli abnormally in autism is not clear. Here, we found that looming-evoked defensive response is impaired in a subgroup of the valproic acid (VPA) mouse model of autism. By combining the conventional neurotracer and transneuronal rabies virus tracing techniques, we demonstrated that synaptic connections in the SC-LP-BLA pathway were abnormal in VPA mice whose looming-evoked defensive responses were absent. Importantly, we further translated the finding to children with autism and observed that they did not present looming-evoked defensive response. Furthermore, the findings of the DTI with the probabilistic tractography showed that the structural connections of SC-pulvinar-amygdala in autism children were weak. The pulvinar is parallel to the LP in a mouse. Because looming-evoked defensive response is innate in humans and emerges much earlier than do social and language functions, the absence of defensive response could be an earlier sign of autism in children.

## Introduction

Rapidly approaching objects, known as visual looming, constitute an intrinsic and unconditional warning cue to elicit automatic defensive response in dealing with emergency situations. It is of crucial importance to the survival of an organism and it can be observed in virtually all animal species, including humans^1–6^. Although there is general agreement that the amygdala is critical for detecting threats and triggering defensive response, there is a debate regarding whether the threat information is transmitted through a visual cortical or subcortical pathway^7^. Converging evidence from animal and human imaging studies provide some fuel in favor of the subcortical pathway. Prior studies in rodents documented the role of midbrain structures for rapid processing of threatening stimuli, supporting an important role of subcortical pathway to amygdala^8,9^. Using looming stimulations that mimic an approaching aerial predator to initiate a rapid escape response^5,10^, a superior colliculus (SC)-lateral posterior nucleus (LP)-basolateral amygdala (BLA) pathway has been revealed for detecting visual threats in mice^9^. That is in agreement with neuroimaging studies in healthy human subjects report co-activation of subcortical structures of the amygdala, the SC, and the pulvinar nucleus of the thalamus during non-conscious visual perception of fearful stimuli^11–14^. Investigations on patients with cortical blindness after V1 lesion provide additional support for the subcortical involvement during non-conscious perception of fearful stimuli^15,16^. Importantly, recent anatomical studies using *in vivo* tractography provided evidence for the existence of the SC-pulvinar-amygdala pathway in non-human primates, healthy humans, and a blindsight patient with destruction of the visual cortex^17–19^.

The dysfunction of the amygdala and SC has been observed in animal models of autism. This dysfunction is thought to be related to deficits in fear processing and attention^20–23^. Clinically, individuals with autism also exhibit a “lack of fear in response to real dangers” and difficulty with reading fearful emotions^24,25^. Furthermore, neuroimaging studies suggested the abnormal activation of the subcortical nucleus, including the amygdala, SC, and pulvinar (equivalent to LP in rodents), when autism patients were viewing fearful stimuli^26–29^. Whether the structure and function of the subcortical pathway regulating looming-evoked defensive response are abnormal in animal model of autism and also in autism patients has not been verified.

This study aims to investigate the subcortical visual pathway, SC-LP-BLA in autism mouse model and SC-pulvinar-amygdala in children with ASD, on modulating looming induced defensive response by combining data from hypothesis-driven animal experiments with tractography data from an association study on a group of children with ASD. The present study investigated only male mice and children with ASD because the epidemiology studies reported a high male-to-female ratio among children diagnosed with autism^30,31^. In the animal study, we first evaluated the looming-evoked defensive response in an autism-like mouse model (VPA mice) by measuring their flee and freeze behaviors. The neuronal mechanism underlying the dysfunction of looming-evoked defensive response in VPA mice was investigated by the conventional neurotracer and transneuronal rabies virus tracing and c-Fos brain mapping. The human study established a modified looming paradigm to assess defensive behavior in ASD children, who were then assigned to a responding and non-responding subgroup according to their behavior evaluations. The SC-pulvinar-amygdala pathway was compared between the responding and non-responding groups using measures of probabilistic tractography. Translating our findings from animals to human clinical situations, we are finally able to provide evidence that the amygdala subcortical pathway is impaired in individuals with ASD, and it could be the underlying mechanism for their deficits in looming induced fear processing and defensive response.

## Methods

### Animals and participants

For the animal study, all experiments were approved by the Jinan University Institutional Animal Care and Use Committee and all methods were performed in accordance with their guidelines and regulations. Young male (3–4 weeks old) C57BL/6 mice were used in this study. Animals were housed in a 12-hour:12-hour light-dark cycle (lights on at 0700) with food and water provided *ad libitum*. Animals were randomly allocated to experimental and control groups. The experimenters were blind to the experimental group, and the order of testing was counterbalanced during behavioral experiments.

For the human study, thirty-three male children with diagnoses of autism participated in the behavioral evaluation of looming stimulation. Clinical diagnoses were confirmed using the Childhood Autism Rating Scale (CARS)^32^ and expert clinical judgment according to the DSM-IV criteria. Children with ASD-related medical conditions (e.g., fragile X syndrome) and other neurological conditions (e.g., epilepsy) were excluded. The developmental quotient (DQ) scores of all participants were assessed using the Gesell Developmental Diagnosis Scale (GDDS)^33^. A subsample of 23 ASD children also participated in the MRI study. Informed consent of study participation was obtained from the parents or guardians of the participants. Informed consent of online open-access publication of identifying information/images in Supplementary Movies 3 and 4 was also obtained from the occupational therapist and parents or guardians of the participants. This study was reviewed and approved by the Jinan University and all methods were performed in accordance with their guidelines and regulations.

### Surgery and intracranial injection

The mice were anesthetized (Avertin, 13 μl/g, intraperitoneally) and placed in a stereotaxic instrument (RWD, Shenzhen, China). Erythromycin eye ointment was applied to prevent corneal drying, and a heat pad (RWD, Shenzhen, China) was used to maintain the body temperature at 37 °C. A small craniotomy hole was made using a dental drill (OmniDrill35, WPI), and a micropipette connected to a Quintessential Stereotaxic Injector (Stoelting, Wood Dale, IL) and its controller (Micro4; WPI, Sarasota, USA) were used for injection.

For disynaptic tracing of the SC → LP → BLA pathway, a 0.4-μl helper virus (rAAV-hSyn-GFP-2a-TVA-2a-RVG-WPRE-pA) (2 × 10^8^ particles/ml) was injected into the LP (6 C57BL/6 mice) (*AP:* −2.4 mm; *ML*: ±1.5 mm; *DV*: −2.2 mm). The pipette was held in place for 10 minutes and then withdrawn slowly. Twenty-one days later, 0.2 μl of SAD-ΔG-DsRed (EnvA) (2 × 10^8^ particles/ml) was injected into the BLA (*AP:* −1.40 mm; *ML:* ±3.25 mm; *DV*: −4.80 mm).

For the retrograde labeling of LP-projecting SC neurons and BLA-projecting LP neurons, CTB Alexa Fluor conjugates (CTB-488, Invitrogen Inc., Grand Island, NY) were injected into the LP (0.1 μl/injection, *AP:* −2.4 mm; *ML*: ±1.5 mm; *DV*: −2.2 mm) and BLA (0.1 μl/injection, *AP:* −1.40 mm; *ML*: ±3.25 mm; *DV*: −4.80 mm), followed by 0.03 μl of oil (sesame oil; Sigma-Aldrich Corp.) to limit the diffusion of the CTB tracer.

Following injection, the wound was sutured, and antibiotics (bacitracin and neomycin) were applied to the surgical wound. Ketoprofen (5 mg/kg) was injected subcutaneously. Animals were allowed to recover from anesthesia under a heat lamp.

### Injection site verification

After transcardial perfusion with 0.9% saline followed by 4% paraformaldehyde in 0.1 M of PBS, the brain was removed and post-fixed with 4% paraformaldehyde overnight at 4 °C. Then, it was transferred into a 30% sucrose solution until sectioning with a cryostat (CM1900, Leica Microsystems, Bannockburn, IL). A series of 40-μm sections were collected for the verification of injection sites. tissue sections were examined under epifluorescence using a Leica DM6000B microscope.

### Image analysis

Brain sections across SC, LP, and BLA were imaged with a Zeiss 700 confocal microscope with 5x or 20x objectives, or a 40x oil immersion objective. Contrast and brightness were adjusted, and the red-green images were converted to magenta-green.

### Nest building test

The ability to build nests was compared between Saline mice and VPA mice as described by Deacon *et al*.^34^. Briefly, mice were individually housed in clear cages for 24 hours prior to the test. Nesting squares (Nestlets) were subsequently provided in each cage. Nest building was assessed based on a five-point scale – 1: Nestlet not noticeably touched; 2: Nestlet partially torn; 3: Nestlet mostly shredded, but no identifiable nest; 4: Identifiable, but flat nest; and 5: A near perfect nest.

### Three-chambered social test

The sociability of Saline mice and VPA mice was measured with the three-chambered social test as described by Peñagarikano *et al*.^35^. Briefly, the test mouse was placed in a closed central chamber (#2) and allowed to acclimate for 10 minutes. Next, a novel strange mouse was placed under an inverted wire cup in chambers #1 or #3, with a duplicate cup placed in the remaining chamber. The gates between the chambers were raised, and the behavior of the mouse was recorded for 10 minutes. The total time spent with the novel strange mouse versus the novel object was analyzed with a two-tailed paired -test for each treatment group. Failure to achieve a statistically significant preference for spending time with a novel stranger was considered indicative of abnormal behavior. A set of novel stranger mice had been previously acclimated to being restrained for 10-minute periods within the confines of the inverted cup. The stranger mice were alternated from side one to side three to ensure that a preference for a specific side by the test mice did not confound the outcome. The apparatus was wiped down with 75% ethanol at the onset of investigations and between changes of mice thereafter.

### Looming stimulation test

For the animal study, the looming stimulation test was performed in a 50 × 50 × 37 cm closed arena. A LED monitor was embedded in the ceiling to present the looming stimulus. The looming stimulus, which consisted of an expanding black disc, appeared at a diameter of a visual angle of 2° to 20° in 0.25 s, and it was presented once in 0.25 s or 15 times in 10.75 s. The experimenter triggered the stimulation manually when the mouse was in the center of the arena.

For the human study, a modified looming paradigm was adopted. Briefly, the participants were tested individually in a darkened room (approximately 5.80 m × 5.50 m, length × width) located in the rehabilitation center of the First Affiliated Hospital of Jinan University, China. The participants were instructed to play with one therapist and were allowed to move freely in the room. The visual stimuli included a rock animation presented on a screen (1.65 m × 1.25 m, length × width) approximately 2 m in front of the participants. The rock animation was approximately 1.20 m × 0.90 m (length × width) and appeared in the center of the screen. Each image loomed toward the children from a visual angle of 2° to 20° in 0.25 s, which mimicked the scenario of a child’s being hit with a falling rock. The image was presented three times with a randomized interval of three to five minutes. Stim software was used to program and run the experimental procedure. The behavior responses to the looming-rock paradigm were simultaneously and independently recorded and evaluated by two observers. One observer is a psychology master student, and another is an occupational therapist, and both received observer training after total agreement was achieved by both observers. The participants were then assigned to one of the two groups according to their performance on behavior evaluation. The responding group involved the participants who displayed defensive behaviors, such as running away, trying to escape, removing themselves (flight), freezing, being immobilized, or hiding. In contrast, the non-responding group demonstrated impaired defensive behaviors; the participants ignored the rock animation and continued playing.

### Optomotor test

Optomotor test were conducted in Saline mice (n = 7 animals) and VPA mice (n = 6 animals). Briefly, Mice were placed on a platform in the form of a grid (12 cm diameter, 19.0 cm above the bottom of the drum) surrounded by a motorized drum (29.0 cm diameter) that could be revolved clock-wise or anticlockwise at two revolutions per minute. After 5 min of adaptation in the light (400 lux), vertical black and white stripes of a defined spatial frequency were presented to the animal. These stripes were rotated alternately clockwise and anticlockwise, for 2 min in each direction with an interval of 30 s between the two rotations. Various spatial frequencies subtending 0.03, 0.13, 0.26, 0.52 and 1.25 cycles/degree were tested individually on different days in a random sequence. Animals were videotaped with an infrared digital video camera for subsequent scoring of head tracking movements.

### MRI data acquisition

MRI scans were acquired on a GE Discovery MR750 3 T system at the First Affiliated Hospital of Jinan University, China. An eight-channel phased-array head coil was used for the imaging experiments. The children were sedated for imaging using oral chloral hydrate (0.8~0.9 ml/kg). A pediatrician trained in MRI procedures was in attendance throughout the examination. The DTI scan consisted of a single-shot diffusion-weighted EPI sequence with the following parameters: TR/TE = 5000/70 ms, matrix = 256 * 256, FOV = 256 mm, slice thickness = 3 mm without gap, number of slices = 46 axial slices, diffusion directions = 25, b-values = 0 and 1000 s/mm2. A 3D-BRAVO sequence was collected for anatomical localization with the following parameters: TR/TE = 8.2/3.2 ms, inversion time (TI) = 450 ms, flip angle = 12°, matrix = 256 * 256, FOV = 240 mm, slice thickness = 1.0 mm without gap.

### Data analysis

For the animal study, data analysis was done by experimenters blind to experimental conditions. One-way analysis of variance (ANOVA) was used to quantify the duration from start to end, the peak speed during and after looming stimulation, and the number of c-Fos + cells and rabies virus/ CTB–labeled neurons. Data are shown as mean ± standard error of the mean (SEM). Statistical significance was set at *p* < 0.05.

Four Saline mice and 4 VPA mice, received BLA injection of rabies virus and LP injection of helper virus, were used to analyze the number of rabies labeled LP neurons and number of BLA-projecting LP neurons. In each mouse, the number of rabies labeled LP neurons and number of BLA-projecting LP neurons were counted in 4 serial brain sections (40 μm/section) across the LP and BLA, respectively. Finally, the average number of rabies labeled LP neurons and BLA-projecting LP neurons was calculated in the 4 mice.

For the human DTI study, image analyses and tensor calculations were carried out by using FSL (http://www.fmrib.ox. ac.uk/fsl/index.html) and tract-based spatial statistics (TBSS)^36,37^. First, an experimenter inspected all volumes for severe motion and other artifacts. Diffusion-weighted images were then corrected for every current distortion and head motion. A brain mask was generated to separate the brain from non-brain areas. The binary brain mask, averaged diffusion-weighted data, b-values, and vector information were then input into FDT, which fits a diffusion tensor model at each voxel. The result of this process was an FA map for each participant. Following this, the probabilistic tractography was performed following the method described previously^38^. Fiber tracking was initiated from all voxels within the seed of ROI in the diffusion space to generate 5000 streamline samples, with a step length of 0.5 mm, a curvature threshold of 0.2, and a maximum number of 2000 steps^39^. All ROI masks of bilateral amygdala, SC, and pulvinar were drawn manually in accordance with anatomical landmarks by an experienced imaging doctor and inspected for accuracy by the investigator. The ROIs were then linearly transformed into the native space of each participant. Fiber tracking was performed between the amygdala and SC via the pulvinar (used as waypoint) in both hemispheres of each individual participant. Fiber tracking was performed in both directions, from the amygdala (seed ROI) to SC (target ROI) and from SC (seed ROI) to the amygdala (target ROI). For each participant, the two tractographies were averaged to produce final tract pathways in each hemisphere. The tracts thresholded at 5% were binarized, transformed to the MNI space, and summed across the participants to produce group probability maps. These group probabilistic maps were set at a threshold that allowed the paths present in only at least 40% of the subjects to be displayed. The John Hopkins University (JHU) white matter tractography atlas was used for tract labeling^40^. Then, the mean FA from voxels within these group probability maps were calculated for each participant and exported to SPSS. We used two-sample t tests to determine significant differences in FA between the different participant groups.

## Results

### VPA mice exhibited deficits in looming-evoked defensive response

**Figure 1:**
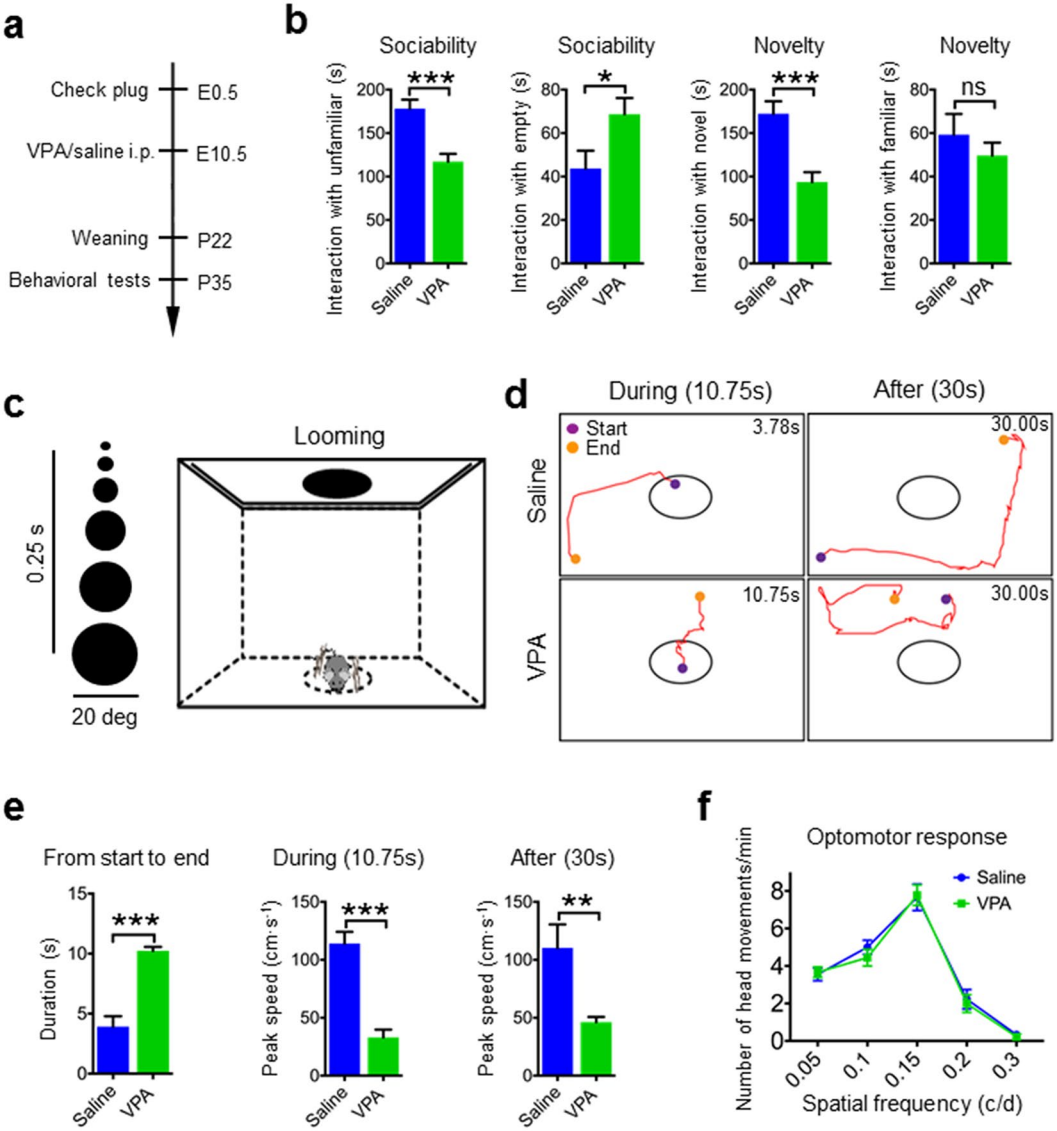
Looming-evoked defensive response was impaired in a subgroup of VPA mice. (**a**) Experimental design of behavior tests in VPA mice. (**b**) VPA mice exhibit increased autism-like behaviors in three-chamber test and next building test. Unpaired t test; *p < 0.05, **p < 0.001, ***p < 0.001; ns = no significant difference. (**c**) Schematic of looming animation and testing arena. (**d**) Representative traces of animal movement for 10.75 s and after 30 s of looming stimulation in Saline mice and VPA mice; time in corner is duration from start to end. (**e**) Duration from start to end points, peak speed during and after looming stimulation in Saline mice and VPA mice. Unpaired t test; **p < 0.001, ***p < 0.0001. (**f**) The optomotor response of animals treated with saline or VPA tested under photopic condition. The responses in both animal groups were similar.

An autism-like mouse model was created by exposing mice to VPA on the 10.5^th^ day of gestation, whereas mice in the control group were treated with saline (Fig. 1a). Compared with the offspring of saline-treated mice (Saline mice), the offspring of VPA-treated mice exhibited increased autism-like behaviors, including a significantly lower score on the nest building test and significantly less contact time with the novel object on the three-chamber test (Fig. 1b).

Looming-evoked defensive responses were tested with a behavioral assay of a rapidly expanding dark disk stimulus (looming stimulus of −2 degrees of the visual angle expanded to 20 degrees in 250 ms) presented overhead 15 times over 10.75 s (Fig. 1c)^5,9,10^. Under this paradigm, 98.3 ± 0.7% of saline-mice responded by fleeing from the center of the arena and freezing (Fig. 1d and Supplementary Movie 1). By contrast, looming stimulation did not evoke a flee or freeze response in 43.9 ± 3.7% of VPA mice, which continued to explore the arena during and after the looming stimulation (Fig. 1d,e and Supplementary Movie 2). In the optomotor test, there was no significant difference between Saline mice and VPA mice (Fig. 1f), indicating that visual ability of VPA mice was unaffected. These results indicate that looming-evoked defensive response is impaired in a subgroup of VPA mice.

### Looming activation of SC-LP-BLA pathway was impaired in VPA mice

**Figure 2:**
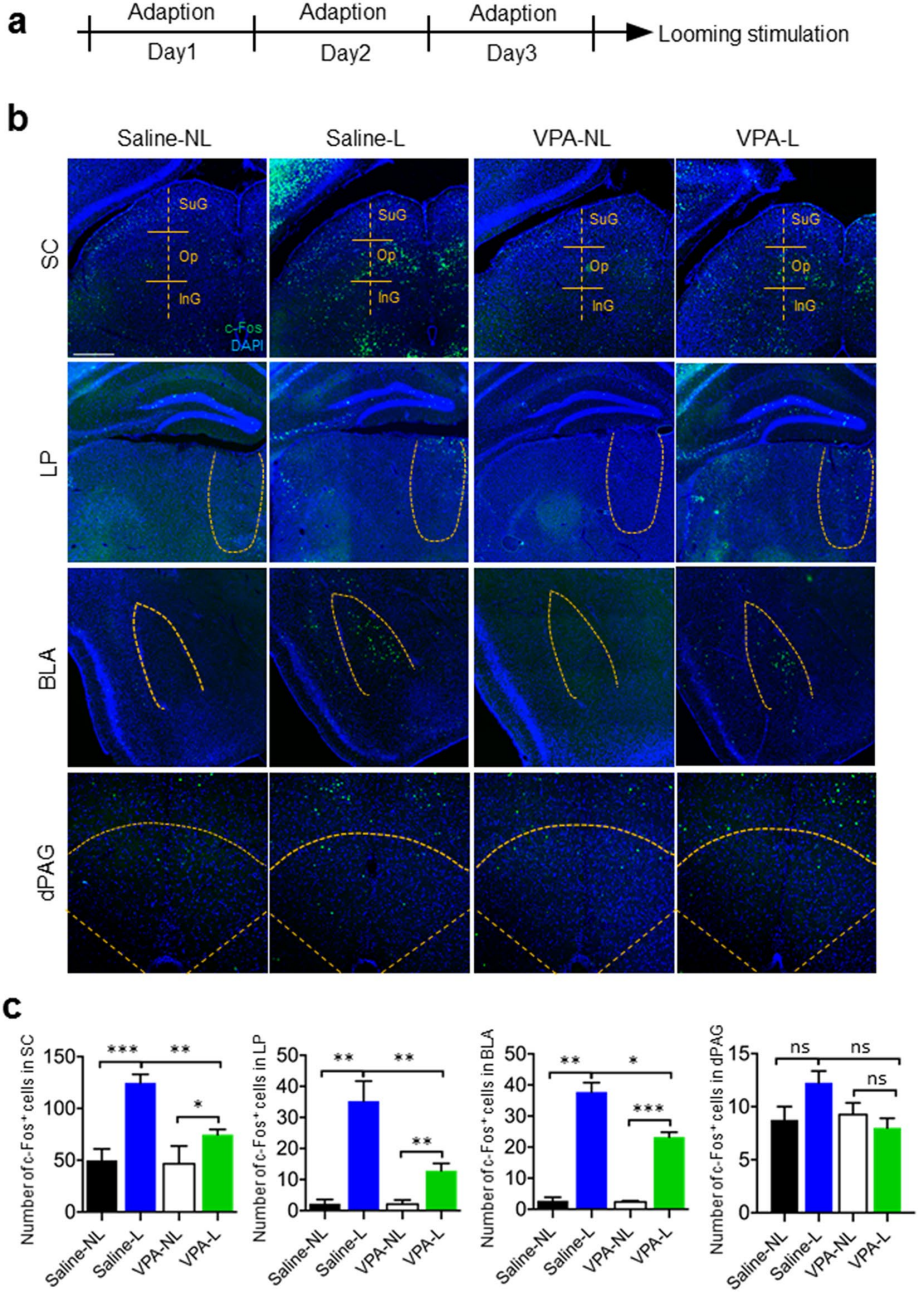
Looming induced c-Fos activation of the superior colliculus-lateral posterior nucleus-basolateral amygdala (SC-LP-BLA) pathway was impaired in non-responding VPA mice. (**a**) Design of looming-evoked c-Fos experiment. (**b**) Illustration of c-Fos+ cells in SC, LP, BLA, and dPAG in saline-NL, saline-L, VPA-NL, and VPA-L groups. Scale bar, 50 μm. (**c**) Quantification of c-Fos+ cells in SC, LP, BLA, and dPAG in each group of animals. One-way ANOVA; ns = no significant difference, *p < 0.05, **p < 0.001, ***p < 0.0001.

The activation of the subcortical circuit from SC → lateral posterior nucleus of the thalamus → basolateral amygdala (SC-LP-BLA) underlies the induction of looming-evoked defensive response^9,10^. To determine whether the looming-evoked activation of the SC-LP-BLA pathway was affected in VPA mice, which had no response to looming stimulation, a looming-related c-Fos mapping strategy was adopted to assess the changes of neuronal activity in the SC-LP-BLA pathway. Twelve Saline mice and thirteen VPA mice were given three days of adaptation in the arena. On the fourth day, a single looming stimulus (15 presentations over 10.75 s) was presented to six Saline mice (Saline-L) and seven VPA mice (VPA-L) (Fig. 2a). The remaining Saline mice (n = 6, Saline-NL) and VPA mice (n = 6, VPA-NL) were treated in a similar manner without the presentation of the looming stimulus. Looming stimulation significantly increased the number of c-Fos positive neurons in the SC-LP-BLA pathway in both Saline mice and VPA mice (Saline-NL vs Saline-L: SC, *p* < 0.0001; LP, *p* < 0.001; BLA, *p* < 0.001. VPA-NL vs VPA-L: SC, *p* < 0.05; LP, *p* < 0.001; BLA, *p* < 0.0001. Fig. 2b,c). However, the number of c-Fos positive neurons in the SC-LP-BLA pathway was significantly decreased in VPA-L animals compared to saline-L animals (Saline-L vs VPA-L: SC, *p* < 0.001; LP, *p* < 0.001; BLA, *p* < 0.05. Fig. 2b,c). In Contrast, there was no significant changes in the number of c-Fos positive neurons in dorsal periaqueductal grey (dPAG), which is considered to play an important role in the regulation of defensive behavior (Fig. 2b,c). These results suggest that c-Fos induction was reduced in VPA mice, which exhibited impaired looming-evoked defensive response.

### Connections of SC-LP-BLA pathway were impaired in VPA mice

**Figure 3:**
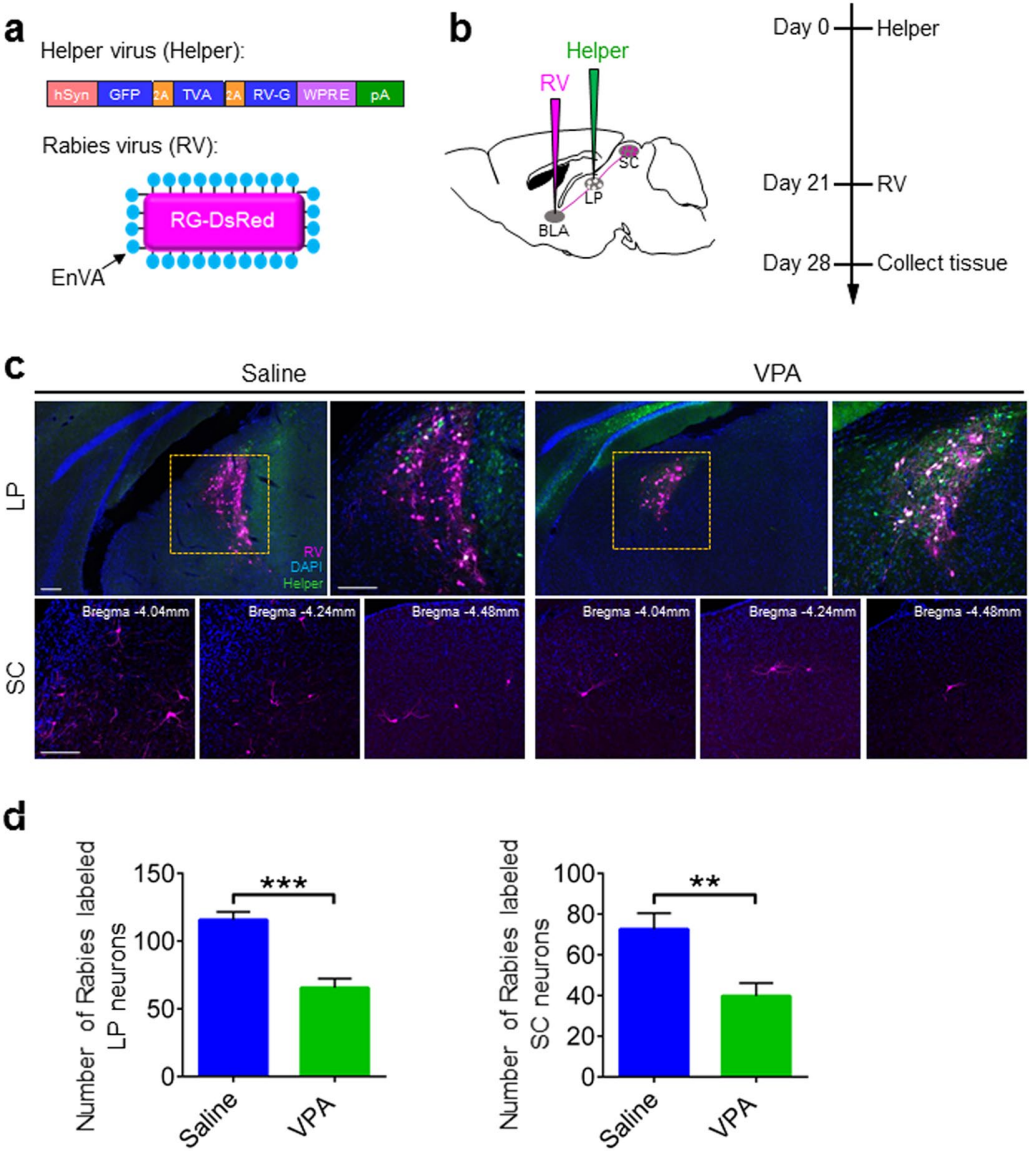
Disynaptic connections from SC to BLA were decreased in non-responding VPA mice. (**a**) Design of helper virus and SAD-ΔG-DsRed (EnvA). (**b**) Experimental design of virus tracing. (**c**) Illustration of LP relay neurons (white) and SC presynaptic neurons in Saline mice and VPA mice. Scale bars, 20 μm. (**d**) Quantification of rabies virus labeled LP neurons and SC neurons in Saline mice and VPA mice. Unpaired t test; **p < 0.001, ***p < 0.0001.

Abnormal synaptic connections in the brain have been found in autism^41,42^. We postulated that the reduced c-Fos induction of the SC-LP-BLA pathway in VPA mice might be accompanied by disrupted synaptic connectivity. To test this hypothesis, we first compared the disynaptic connections between SC and BLA in Saline mice and VPA mice by using a modified rabies transsynaptic tracing method (Fig. 3a). LP neurons were infected by AAV expressing the rabies glycoprotein and histone-tagged green fluorescent protein (helper) (Fig. 3a,b). SAD-ΔG-DsRed (EnvA) was injected into BLA to infect helper+ BLA-projecting LP neurons via their presynaptic terminals (Fig. 3a,b). The double-infected rabies-DsRed+/Glyco-GFP+ LP relay neurons produce infectious ΔG-rabies-DsRed, which propagates transneuronally to infect the SC neurons that have formed synapses with them (Fig. 3c). Compared with Saline mice, the number of rabies viruses labeled SC neurons and LP neurons was significantly decreased in VPA mice (*p* < 0.001, Fig. 3c,d). These results suggest that the disynaptic connections from SC to BLA were abnormally decreased in VPA mice.

**Figure 4:**
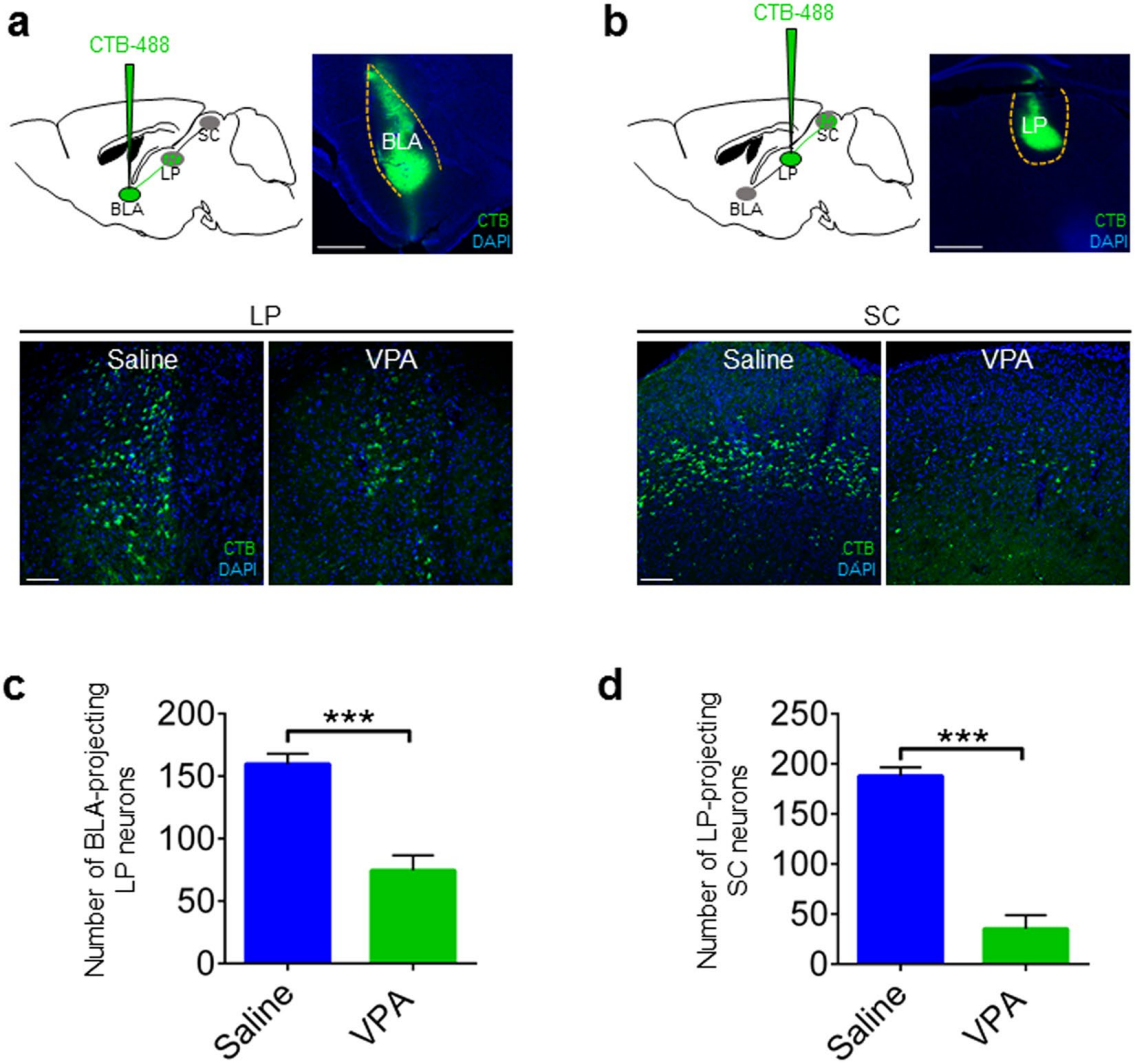
Synaptic connections from SC to LP and from LP to BLA were impaired in non-responding VPA mice. (**a**) Illustration of CTB-labeled BLA-projecting LP neurons in Saline mice and VPA mice in a retrograde manner. Scale bars, 20 μm. (**b**) Illustration of CTB-labeled LP-projecting SC neurons in Saline mice and VPA mice in a retrograde way. Scale bars, 20 μm. (**c**,**d**) Quantification of CTB + BLA-projecting LP neurons and LP-projecting SC neurons in Saline mice and VPA mice. Unpaired t test; ***p < 0.0001.

Next, retrograde tracing was conducted to determine whether the connections of LP-BLA or SC-LP or were impaired in VPA mice. First, to label BLA-projecting LP neurons in a retrograde way, 100 nl of the cholera toxin B subunit conjugated to Alexa Fluor 488 (CTB-488) was injected into BLA (Fig. 4a). The number of CTB-488-labeled BLA-projecting LP neurons was significantly decreased in VPA mice compared to Saline mice (*p* < 0.0001, Fig. 4a,c). Next, to label LP-projecting SC neurons in a retrograde way, 100 nl of CTB-488 was injected into LP (Fig. 4b). The number of CTB-488-labeled LP-projecting SC neurons was also significantly decreased in VPA mice compared to Saline mice (*p* < 0.0001, Fig. 4b,d). These results suggest that the synaptic connections from LP to BLA and from SC to LP are disrupted in VPA mice.

### Children with autism exhibited deficits in looming-evoked defensive response

**Figure 5:**
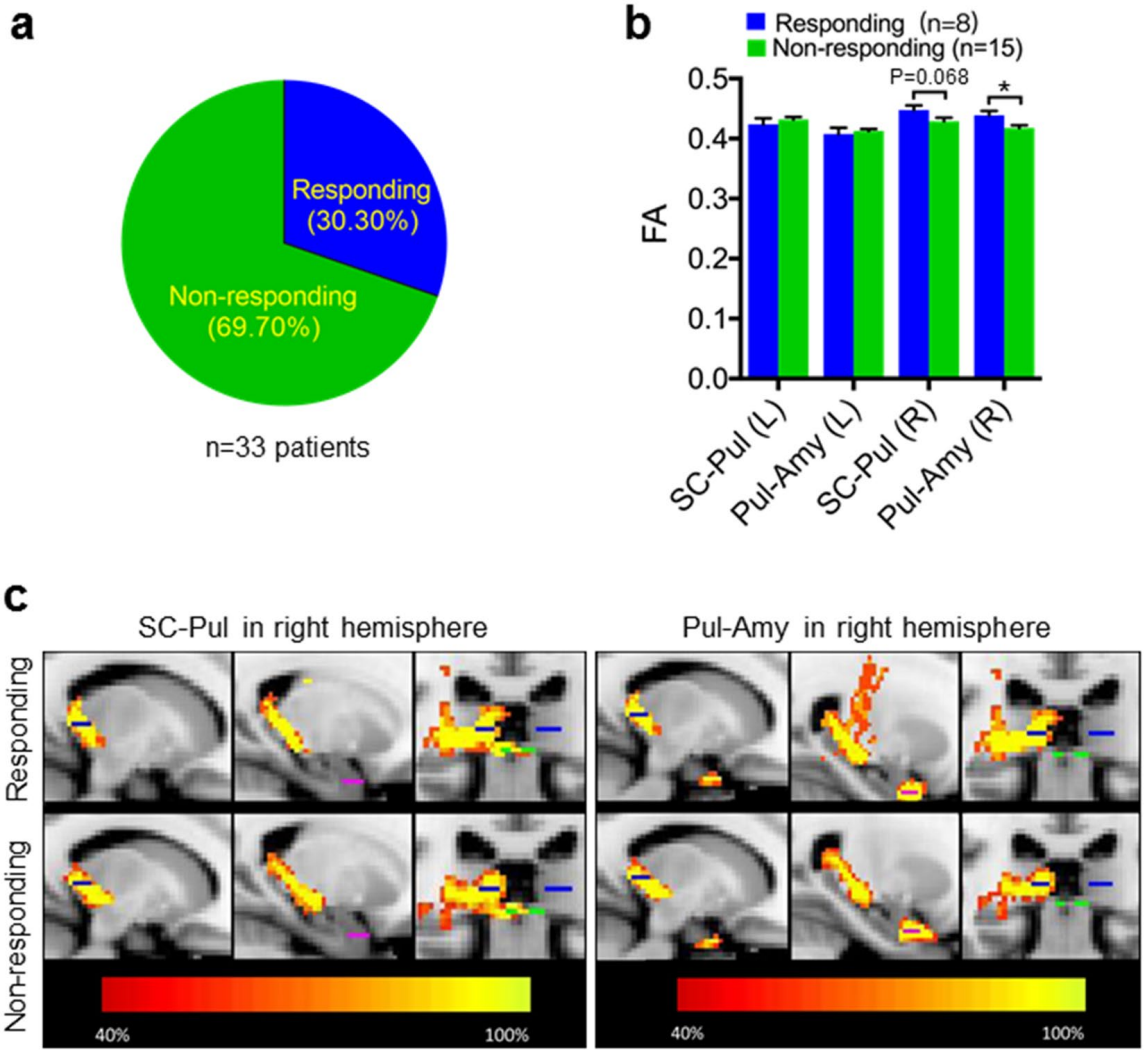
Looming-evoked defensive response was impaired in a subgroup of children with autism accompanied by abnormal synaptic connections in SC-pulvinar-amygdala pathway. (**a**) Looming-evoked defensive responses were impaired in 69.70% of children with autism. (**b**) Higher FA values were revealed for right SC-pulvinar and right pulvinar-amygdala in the responding group (n = 8) compared with non-responding group (n = 15). Unpaired t test; *p < 0.05. (**c**) Within-group probabilistic tractography maps for right SC-pulvinar and right pulvinar-amygdala.

Looming-evoked defensive response was tested in thirty-three male children with autism by using an adapted looming paradigm with a rapidly expanding rock stimulus (looming stimulus of −2 degrees of the visual angle expanded to 20 degrees in 250 ms) presented to the child three times with a randomized interval of three to five minutes. Ten of the children with autism (30.30%) displayed defensive behaviors, such as running away, trying to escape, removing themselves (flight), freezing, being immobilized, or hiding (Fig. 5a and Supplementary Movie 3). In contrast, twenty-three of these autism children demonstrated impaired defensive behaviors in terms of ignoring the rock animation and continued playing (Fig. 5a and Supplementary Movie 4). The children with autism in the responding and non-responding groups were matched by their ages, DQ, and scores on the CARS (Table 1). These results suggest that looming-evoked defensive response was also impaired in a subgroup of children with autism.

**Table 1:** Demographic data for the children with autism in the behavior study and DTI study.

	Behavior study			DTI study		
	Responding (n = 10)	Non-responding (n = 23)	*p*	Responding (n = 8)	Non-responding (n = 15)	*p*
Age(months)	43.80	46.09	0.723	43.38	44.47	0.879
DQ score	51.12	50.41	0.885	59.90	54.49	0.314
CARS score	32.50	32.35	0.863	30.50	31.53	0.346

### FA values of SC-pulvinar and pulvinar-amygdala were reduced in non-responding children with autism

A subgroup of children with autism (n = 23) was also invited to participate in the DTI study (Table 1). In humans, the SC-pulvinar-amygdala subcortical pathway has been found to play an important role in mediating fear-related behaviors^12–15^. Notably, pulvinar is commonly regarded as a LP-related structure in rodents^43^. To examine the connectivity of the SC-pulvinar-amygdala pathway in children with autism, the probabilistic tractographies of SC-pulvinar and pulvinar-amygdala, indexed by fractional anisotropy (FA) values, were compared between responding (n = 8) and non-responding (n = 15) children with autism. The independent t tests revealed higher FA values in the responding group compared with the non-responding group for the right SC-pulvinar (t_1,21_ = 1.923, *p* = 0.068) and right pulvinar-amygdala (t_1,21_ = 2.637, *p* = 0.015) (Fig. 5b,c). The results suggest that there is an under-connectivity of the SC-pulvinar-amygdala pathway in non-responding children with autism.

## Discussion

Among the most critical of the visual functions is the detection of threats in the environment. Rapidly approaching objects, known as looming objects, have been shown to trigger defensive responses across animal species. Lacking the ability to respond to looming stimuli would result in excessive risk-taking and dangerous behavioral consequences. Abnormal defensive behaviors in response to threatening stimuli have been found in mice model of autism^21^ as well as in children with autism^24,25^. In this study, we found abnormal looming-evoked defensive response in both an autism-like mice model and patients with autism. We established an autism-like mice model via a single injection of VPA to pregnant mice on gestational day 10.5. Previous studies found that VPA treatment induced an abnormal differentiation of the neural tube in mice and an increase in autism-like behaviors in their offspring^44,45^. Consistently, we observed deficits of social behaviors in VPA mice through several behavioral tests. The results of the looming test indicated that 43.9 ± 3.7% of the VPA mice failed to freeze or engage in flight in response to looming stimuli. In parallel, the non-response rate for looming stimulation is 76.7 ± 4.3% in children with autism.

Recent studies conducted in mice have justified that looming-evoked defensive response is triggered by the activation of a subcortical pathway consisting of SC, LP, and BLA^9,10^. Following this line of thought, we speculated that the looming-evoked activation of the SC-LP-BLA pathway might be impaired in non-responding VPA mice. This was born out of our c-Fos mapping experiments, which showed that looming-evoked c-Fos expression in SC, LP, and BLA was significantly decreased in non-responding VPA mice compared to responding VPA mice and Saline mice. Disrupted neural connections were observed in autism-like animal models^46–48^. The deficits of the looming-evoked activation of the SC-LP-BLA pathway observed in non-responding VPA mice might be due to disrupted neural connections in the SC-LP-BLA pathway. Consistent with this speculation, results from rabies virus disynaptic tracing suggested an under-connectivity between SC and BLA in non-responding VPA mice. The results of the CTB retrograde labeling further demonstrated that, relative to the controls, the number of LP-projecting SC neurons and BLA-projecting LP neurons was significantly lower in the non-responding VPA mice. It has been well established that the transduction of CTB relies on intact neural connections^49^. Thus, neural connections from SC to LP and from LP to BLA are impaired in non-responding VPA mice.

An SC-pulvinar-amygdala subcortical pathway also exists in humans, in which pulvinar is commonly regarded as a LP-related structure in rodents. Neural connectivity in this pathway may be abnormal in children with autism who have exhibited impaired looming-evoked defensive response. In agreement with this, our DTI data revealed a lower FA in SC to pulvinar and in pulvinar to amygdala in the right hemisphere in non-responding children with autism compared with responding children. Several DTI studies have reported reduced FA in the amygdala and thalamus in patients with autism^50,51^. FA is the most commonly used measure of white matter integrity. A lower FA indicates the breakdown of white matter integrity, which could cause neural noise and disruptions of the conduction of action potentials, finally resulting in less efficient neural transmissions^52,53^. The abnormality of the connectivity in the right SC-pulvinar-amygdala may affect the function of this subcortical pathway. By contrast, the between-group difference of FA values is not significant in the left SC-pulvinar-amygdala pathway. The structural asymmetries concur with the right-hemisphere phasic alertness network comprising the right amygdala, thalamus, dorsolateral and ventrolateral frontal cortices, and anterior cingulate cortex^54^. Importantly, it is consistent with results of an fMRI study on auditory looming^55^. The study presented rising intensity sounds (auditory looming) to healthy subjects and measure their brain activity using fMRI. The results found activations of the right amygdala, but not the left amygdala^55^.

To our knowledge, this study is the first to confirm an impairment of looming-evoked defensive response in both an autism-like animal model and autism patients. Our data suggest that autism is associated with abnormal subcortical connectivity, which may lead to the impairment of defensive response. The looming paradigm seems sensitive to marking a subgroup of autism children, suggesting that the looming paradigm may be useful for application in clinical practice for autism screening. Genetic studies found susceptibility genes in ASD, which are known to be important for neural circuit formation—in particular, axonal and dendritic growth, synaptogenesis, and synaptic homeostasis^56–58^. Further studies will be required to determine the genetic mechanism underlying impaired looming-evoked defensive response in autism.

## Electronic supplementary material

Supplementary Movie 1

Supplementary Movie 2

Supplementary Movie 3

Supplementary Movie 4
